# Global trends of Vaccinia oncolytic virus therapy over the past two decades: Bibliometric and visual analysis

**DOI:** 10.3389/fimmu.2023.1063548

**Published:** 2023-02-02

**Authors:** Liu Bo, Liu Tianming, Fan Fengliang, Liang Wenping, Han Jinzuo, Xue Dongbo, Ma Biao, Sun Haijun

**Affiliations:** Department of General Surgery, The First Affiliated Hospital of Harbin Medical University, Harbin, Heilongjiang, China

**Keywords:** bibliometric, knowledge map, VOSviewer, tumor, oncolytic virus

## Abstract

**Background:**

In recent years, the vaccinia oncolytic virus has entered the clinical trial stage of examination and shown good progress. It has many advantages, such as good safety, high oncolytic efficiency, and the regulation ability of the tumor microenvironment, and is expected to be successfully used in the clinical treatment of tumors in the future. However, no bibliometric analysis has so far been performed that generalizes horizontally across this field. Therefore, this study aims to assess the research status and trends in this field from a global perspective to help guide future research priorities.

**Methods:**

In this study, the literature related to vaccinia oncolytic virus published in English on Web of Science from 2002 to 2022 was retrieved, and the bibliometric indicators were analyzed using the Histcite. Pro 2.0 tool, while VOSviewer was used to visualize the research trends and hotspots in this field.

**Results:**

In total, 408 related studies were included. In the past 20 years, the number of related publications in this field has increased year by year, and breakthroughs were made in this field in 2008 and 2013. The research has grown rapidly since 2008, and will likely continue to expand in the years to come. The United States plays a leading role in this area. “MOLECULAR THERAPY-ONCOLYTICS”, “MOLECULAR THERAPY” and “JOURNAL OF TRANSLATIONAL MEDICINE” are core journals that publish high-quality literature on the latest advances in the field. Some authors with numerous high-quality publications include Bell JC and Szalay AA. At present, the research hotspot in this field focus on the clinical application of vaccinia oncolytic virus.

**Conclusion:**

Overall, the number of vaccinia oncolytic virus-related studies is growing rapidly, in relation to which the United States is the most influential country. The clinical application of vaccinia oncolytic virus will affect the crucial development of future research.

## Background

Cancer is one of the leading causes of death and public health problems in both developed and developing countries (1). Worldwide, approximately 7 million people die each year from malignant tumors, and this number is expected to increase to 12 million by 2030 (2). The current tumor treatment options are mainly surgery, chemotherapy and radiotherapy (3). Although these treatments have good therapeutic effects, surgical treatment may cause incomplete tumor removal, surgery-related complications, anesthesia-related complications, etc. Risks arise in radiotherapy, although it offers the advantages of precision, speed and convenience. However, excessive radiotherapy may cause tissue fibrosis and has a poor effect on metastatic tumors. In terms of chemotherapy, although the research and development of tumor drugs accounts for a quarter of total drug research and development, the number of specific tumor-targeted drugs is currently small (4). Therefore, it is very important to find a tumor treatment method that can specifically kill tumors, has fewer complications, and is highly safe.

Oncolytic virus is a potential gene therapy drug that operates *via* its specific replication in tumor cells without harming normal tissue cells, leading to the mechanism of tumor cell lysis and death. Adenovirus (Adv), herpes simplex virus (HSV) and vaccinia virus (VV) have been widely used in the treatment of refractory and relapsed solid tumors (5). Oncolytic viruses have three specific abilities: targeting lysis of tumor cells; enhancing immune responses around tumor cells; replicating and spreading in tumor cells. This allows them to not only directly infect tumor cells, replicate in tumor cells, and cause lysis in tumor cells after growing in the tumor tissue, but also to further enhance tumor killing by mediating the immune system and causing tumor blood vessel shrinkage (6, 7). Among these oncolytic viruses, vaccinia oncolytic virus can directly lyse tumor cells due to its fast replication cycle, high transduction efficiency, broad-spectrum infectivity and tumor tropism. Meanwhile, the vaccinia virus, with a larger coding capacity, has a stronger ability to encode immunostimulating transgenes, and can reshape the tumor immune microenvironment. It also has the ability to induce effective immune responses, and a high safety level for human application (8–10).

Bibliometrics is a common method for systematically reviewing a research field in medicine. Through quantitative analysis of the existing literature, it can not only describe the development trend of a research field, but also systematically analyze the research progress of different countries, institutions, authors, and disciplines, evaluate the research quality of related journals, and use an intuitive map to predict the future development direction of the field, providing guidance for researchers’ next decision-making steps. With the development of genetic engineering and bioinformatics, gene drug therapy for tumors has emerged. Although there are many review articles on the oncolytic virus and gene therapy, the bibliometric research on the vaccinia oncolytic virus is still lacking. We used VOSviewer and Histcite, two bibliometric analysis tools, to analyze and summarize the literature related to vaccinia oncolytic virus, seeking the next hotspots in related fields.

## Materials and method

### Data source

We searched on 2022-04-01 using the Web of Science platform (11, 12), which includes the SCI-EXPANDED, SSCI, AHCI, CPCI-S, CPCI-SSH, BKCI-SSH, ESCI, CCR-EXPANDED, and IC databases.

### Inclusion criteria

(1) Setting the time period to 2002 to 2022. (2) Restriction of the paper type to “article”. (3) Setting the search formula to ALL=(Vaccinia oncolytic virus) AND ALL=(tumor).

### Data extraction and analysis

We selected references whose records show full records and citations, and exported the data retrieved from the Web of Science platform into the “TXT” format. Descriptive statistical analysis was performed using Histcite Pro 2.1. The analysis indicators included the total number of published articles, the year of publication, the country of publication, the publishing institution, the published journal, the author, the keywords, the total number of citations, the average number of citations, and the H-index. Microsoft Excel 2019 was used for data calculation and charting. VOSviewer (version 1.6.18) was used for co-authoring, co-citation, and co-occurrence analysis of countries, institutions, authors, keywords, and scientific knowledge maps. Institutional Review Board (IRB) approval and written consent were not required for this bibliometric analysis.

## Result

### Time of publication and citations

**Figure 1:**
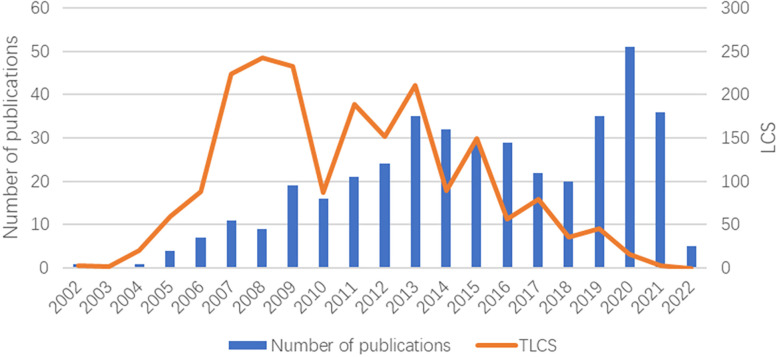
The number of publications and LCS by year over the past 20 years.

A total of 408 related documents in the field of oncolytic viruses and tumors from 2002 to 2022 were collected. The total number of citations was 11,772, the total number of local citations (LCS) was 1,986, and the total number of local citations that were not self-citations (LCSx) was 1,144. The average local citation size (LCS) was 4.87 and the average (LCSx) citation size was 2.80. **Table 1** lists the relevant information regarding the top ten items of literature ranked by LCS. In terms of publication time, in the past 20 years, the number of publications has shown an overall upward trend, indicating that this field has been a research hotspot in recent years. Since the two peaks of LCS in 2008 and 2013, the local citation rate (LCS) has declined, which indicates that although the number of published articles in this field has been increasing in recent years, there may be bottlenecks in some aspects, which have not yet been overcome. In the future, research in this area should focus on innovation (**Figure 1**).

**Table 1:** The relevant information for the top ten items of literature ranked by LCS.

Title	First Author	Journal	Lcs	Gcs
Use of a targeted oncolytic poxvirus, JX-594, in patients with refractory primary or metastatic liver cancer: a phase I trial	Park BH	LANCET ONCOLOGY	103	368
Eradication of solid human breast tumors in nude mice with an intravenously injected light-emitting oncolytic vaccinia virus	Zhang Q	CANCER RESEARCH	102	183
Intravenous delivery of a multi-mechanistic cancer-targeted oncolytic poxvirus in humans	Breitbach CJ	NATURE	84	367
Randomized dose-finding clinical trial of oncolytic immunotherapeutic vaccinia JX-594 in liver cancer	Heo J	NATURE MEDICINE	83	484
Rational strain selection and engineering creates a broad-spectrum, systemically effective oncolytic poxvirus, JX-963	Thorne SH	JOURNAL OF CLINICAL INVESTIGATION	74	160
Systemic armed oncolytic and immunologic therapy for cancer with JX-594, a targeted poxvirus expressing GM-CSF	Kim JH	MOLECULAR THERAPY	59	210
The Oncolytic Poxvirus JX-594 Selectively Replicates in and Destroys Cancer Cells Driven by Genetic Pathways Commonly Activated in Cancers	Parato KA	MOLECULAR THERAPY	46	157
Regression of human pancreatic tumor xenografts in mice after a single systemic injection of recombinant vaccinia virus GLV-1h68	Yu YA	MOLECULA CANCER THERAPEUTICS	38	81
First-in-man Study of Western Reserve Strain Oncolytic Vaccinia Virus: Safety, Systemic Spread, and Antitumor Activity	Zeh HJ	MOLECULAR THERAPY	38	85
Targeted inflammation during oncolytic virus therapy severely compromises tumor blood flow	Breitbach CJ	MOLECULAR THERAPY	37	206

### Contributions of countries

**Figure 2:**
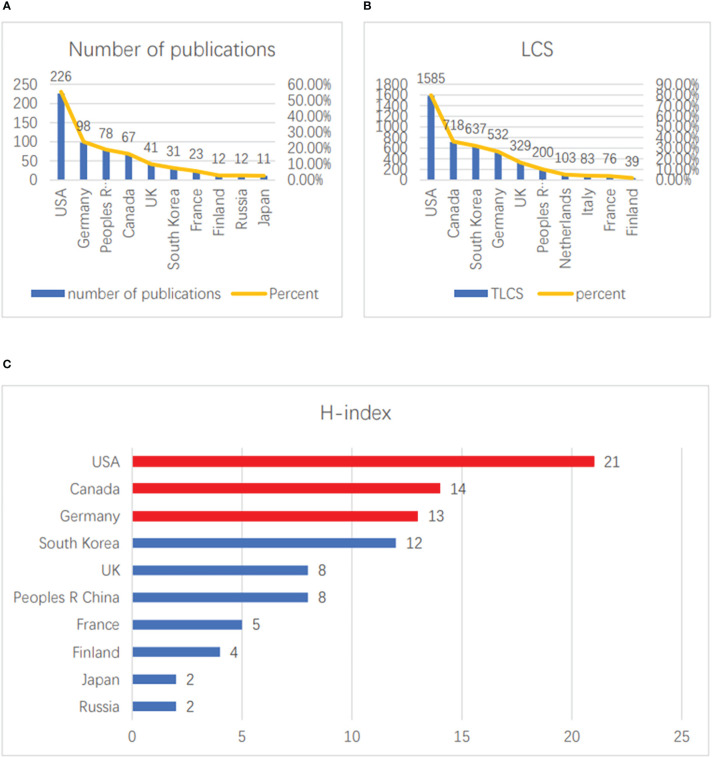
Contributions of countries. **(A)** The top 10 countries with the greatest numbers of publications. **(B)** The top 10 countries with the greatest numbers of LCS. **(C)** The top 10 countries with the highest H-index scores.

For the vaccinia oncolytic virus and tumor-related fields, the top 10 countries with the most publications are shown in **Figure 2A**. Among them, the United States has offered the largest number of publications, with a total of 226, accounting for 55.4% of the total; this is followed by Germany (98, accounting for 24.0% of the total), and China ranks third (78, accounting for 19.1% of the total). The top three countries published more than 90% of the total number of publications in the field. In terms of LCS, the United States has the highest citation rate, at 1,585, accounting for 79.8% of total citations. The second and third countries are Canada (624, 79.8%) and South Korea (597, 36.2%) (**Figure 2B**). In terms of H-index ranking, the United States (21), Canada (14) and Germany (13) are the top three, respectively (**Figure 2C**). All in all, the U.S. has maintained its dominance in this research area in terms of both quantity and quality of publications.

### Contributions of scholars

**Figure 3:**
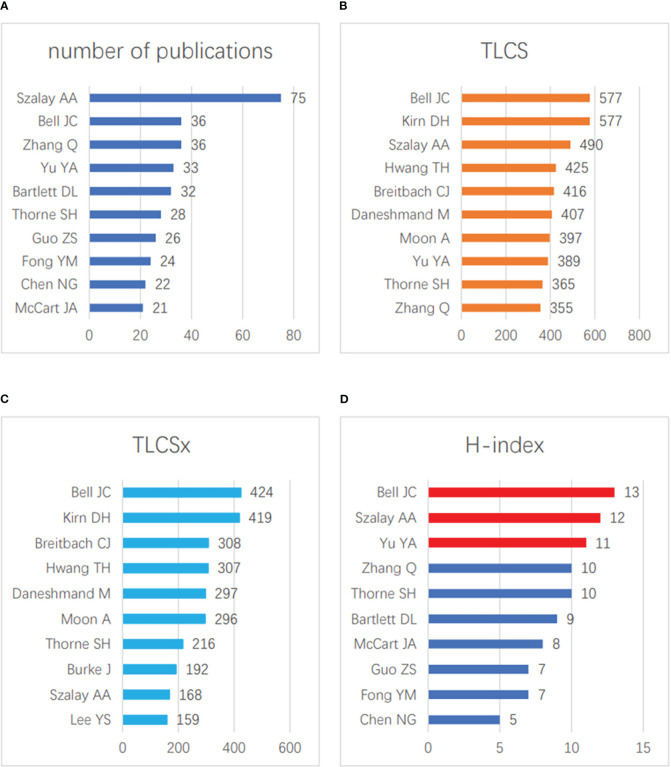
Contributions of scholars. **(A)** The top 10 scholars in terms of the total number of published articles. **(B)** The top 10 scholars in terms of the total number of LCS. **(C)** The top 10 scholars in terms of the total number of LCSx. **(D)** The top 10 scholars with the greatest H-index scores.

In the field of vaccinia oncolytic virus and tumors, the three authors with the highest numbers of published papers were Szalay AA (75 papers), Bell JC (36 papers), and Zhang Q (36 papers) (**Figure 3A**). The three authors with the most total citations were Bell JC (577 times), Kirn DH (577 times) and Szalay AA (490 times) (**Figure 3B**), while the three authors with the most total citations after self-citation were Bell JC (424 times), Kirn DH (419 times), Breitbach CJ (308 times) (**Figure 3C**). In terms of the H-index, Bell JC ranked first (13), followed by Szalay AA (12) and Yu YA (11) (**Figure 3D**). It can be seen that the research of Bell JC and Szalay AA has been widely recognized by scholars in this field.

### Contributions of institutions

**Figure 4:**
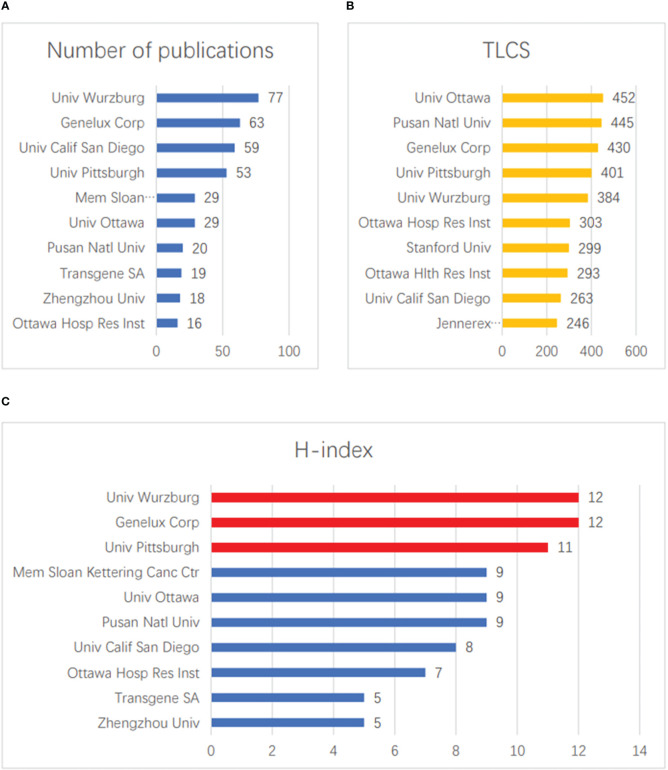
Contributions of institution. **(A)** The top 10 institutions in terms of total publication volume. **(B)** The top 10 institutions in terms of total LCS. **(C)** The top 10 institutions with the largest H-index scores.

The top ten institutions in terms of total publication volume are shown in **Figure 4A**. The three institutions that published the most papers were Univ Wurzburg (77), Genelux Corporation (63), and Univ Calif San Diego (59). The three institutions with the highest numbers of LCSs were Univ Ottawa (452), Pusan Natl Univ (445), and Genelux Corp (430) (**Figure 4B**). In terms of H-index, Univ Wurzburg (12) and Genelux Corporation (12) tied for first place, followed by Univ Pittsburgh (11) (**Figure 4C**).

### Published journals

**Figure 5:**
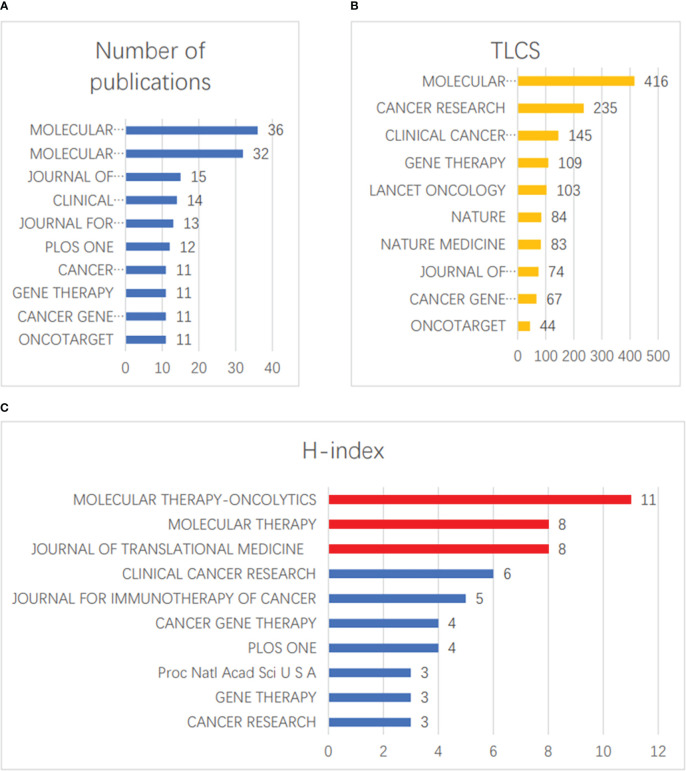
Published journals. **(A)** The top 10 journals with the greatest numbers of publications. **(B)** The top 10 journals with the most LCS. **(C)** The top 10 journals with the greatest H-index scores.

In the field of vaccinia oncolytic virus and tumors, there were 166 relevant articles in the top 10 journals, accounting for 40.69% of the total number of studies. The three journals with the highest numbers of published papers were MOLECULAR THERAPY-ONCOLYTICS (36 papers), MOLECULAR THERAPY (32 papers), and JOURNAL OF TRANSLATIONAL MEDICINE (15 papers) (**Figure 5A**). The three journals with the most LCS were MOLECULAR THERAPY (416 hits), CANCER RESEARCH (235 hits), and CLINICAL CANCER RESEARCH (145 hits) (**Figure 5B**). MOLECULAR THERAPY-ONCOLYTICS (11), MOLECULAR THERAPY (8) and JOURNAL OF TRANSLATIONAL MEDICINE (8) ranked as the top three in terms of H-index (**Figure 5C**).

### Research status and development trend

**Figure 6:**
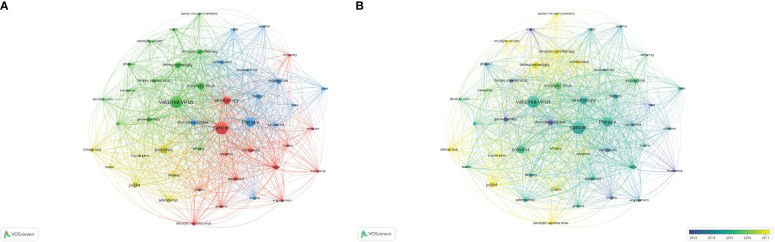
Research status and development trend. **(A)** The 47 keywords that arose more than 16 times were divided into three clusters, represented by different colors. The sizes of the nodes represent the frequency of occurrences. **(B)** Visualization of keywords according to the average publication year. Keywords in yellow appeared later than those in blue (color figure online).

The VOSviewer software was used to set the minimum frequency of keyword occurrences to 16. Among the 1568 keywords, a total of 47 were screened out, and a visual map was drawn for these 47, as shown in **Figure 6A**. All the keywords were divided into four clusters, representing the four different directions of vaccinia oncolytic virus and tumor therapy research. The red part concerns studies of the biological behavior and activated immunity of vaccinia oncolytic virus in tumor cells; the green part concerns studies on the changes of tumor microenvironment after vaccinia oncolytic virus infection; the blue part concerns the treatment of tumors using vaccinia oncolytic virus. As regards the research using animal experiments, the yellow part is the research direction of the clinical application of vaccinia oncolytic virus in the treatment of tumors.

In time trend chart (**Figure 6B**), the color changes from blue to yellow, representing a change in research direction. The early stage was mainly represented by animal experiments on vaccinia oncolytic virus treatment of tumors; in the middle stage, it transitioned to the clinical treatment of tumors with vaccinia oncolytic virus. The current research direction is towards clinical application and treatment, and the research hotspots include jx-594 (a genetically modified vaccinia virus), the tumor microenvironment, suicide genes, and improved designs or combinations of drugs that combine other substances with oncolytic viruses.

### Co-cited references and co-cited scholars

Among the 10,812 cited articles, the minimum number of citations of cited articles was limited to 25, and a total of 44 articles were screened out. VOS software was used to analyze the co-citation intensity of these 44 articles and draw a visual map. The above references are divided into three clusters, and the research topics represented by each the cluster influence each other, and are interdependent. The red cluster concerns those mainly focused on the application of genetic engineering to construct oncolytic vaccinia virus; the main research direction of the green cluster is the clinical treatment of vaccinia oncolytic virus; the main research direction of the blue cluster is using animal experiments to research GLV-1h68.

**Figure 7:**
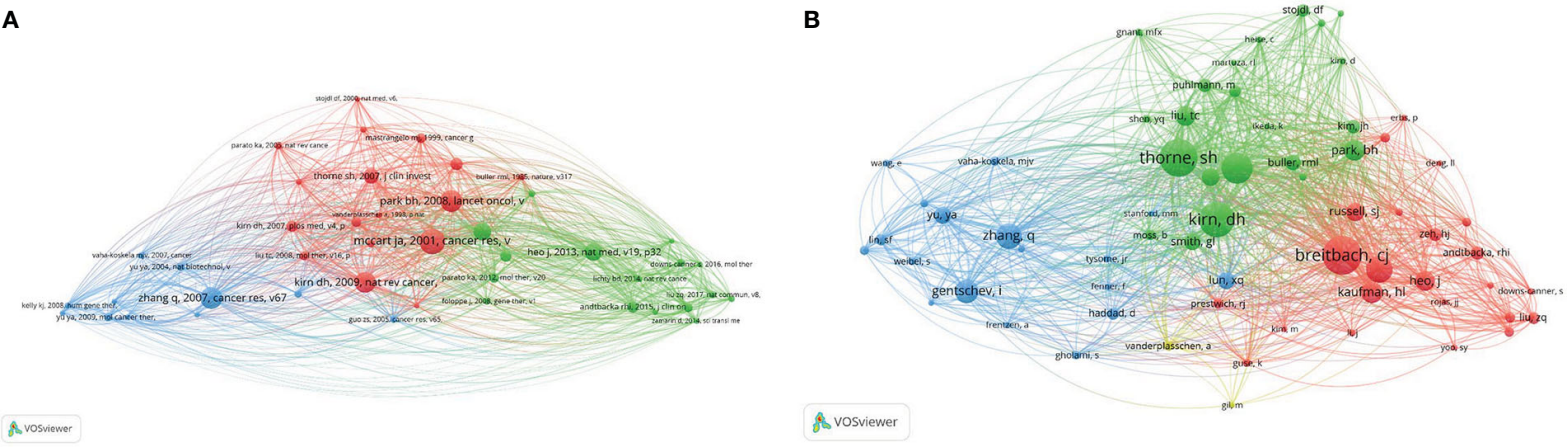
Co-cited references and co-cited scholars. **(A)** Network map of co-cited references. Of the 10,812 references, 44 (classified into three clusters) had been cited at least 25 times. **(B)** Network map of co-cited scholars. Of the 8,405 scholars, 65 (classified into three clusters) had been cited at least 25 times.

For the 8,405 scholars, the minimum number of citations was set at 25, and a total of 65 scholars were screened and included in the analysis. As shown in **Figures 7A, B**, the sizes of the nodes represent the frequency of author citations, among which the research of Breitbach CJ, Kirn DH and Thorne SH is the most cited. In addition, in the figure, clusters with the same color represent scholars with similar research directions. The different research directions are not isolated, and there is mutual influence and close connection between clusters.

### Co-authoring relationship

**Figure 8:**
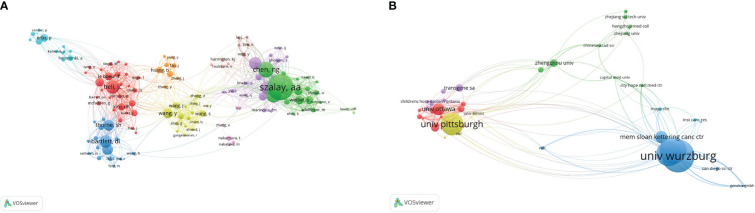
Co-authoring relationship. **(A)** Network map of scholars’ co-authoring relationships. Of the1,765 scholars, 129 showed a co-authoring relationship. **(B)** Network map of institutional co-authoring relationship. Of the 5,476 institutions, 50 showed a co-authoring relationship.

For co-authorship relationships, the minimum number of papers published by authors was limited to 5, and 132 authors were screened out of the 1,765 authors, of which 129 authors had co-authorship relationships (**Figure 8A**). The sizes of the nodes in the graph represent the number of articles published by the authors, and the connections between the nodes represent the cooperative relationship between said authors. We found that collaboration among scholars is closely related to nationality, and authors in the same cluster are mostly from the same country.

In the institutional co-authorship relationship, the minimum number of publications by an institution was limited to 5,476, and 55 of them finally met this standard. Of these, 50 institutions were linked to each other (**Figure 8B**).

**Figure 9:**
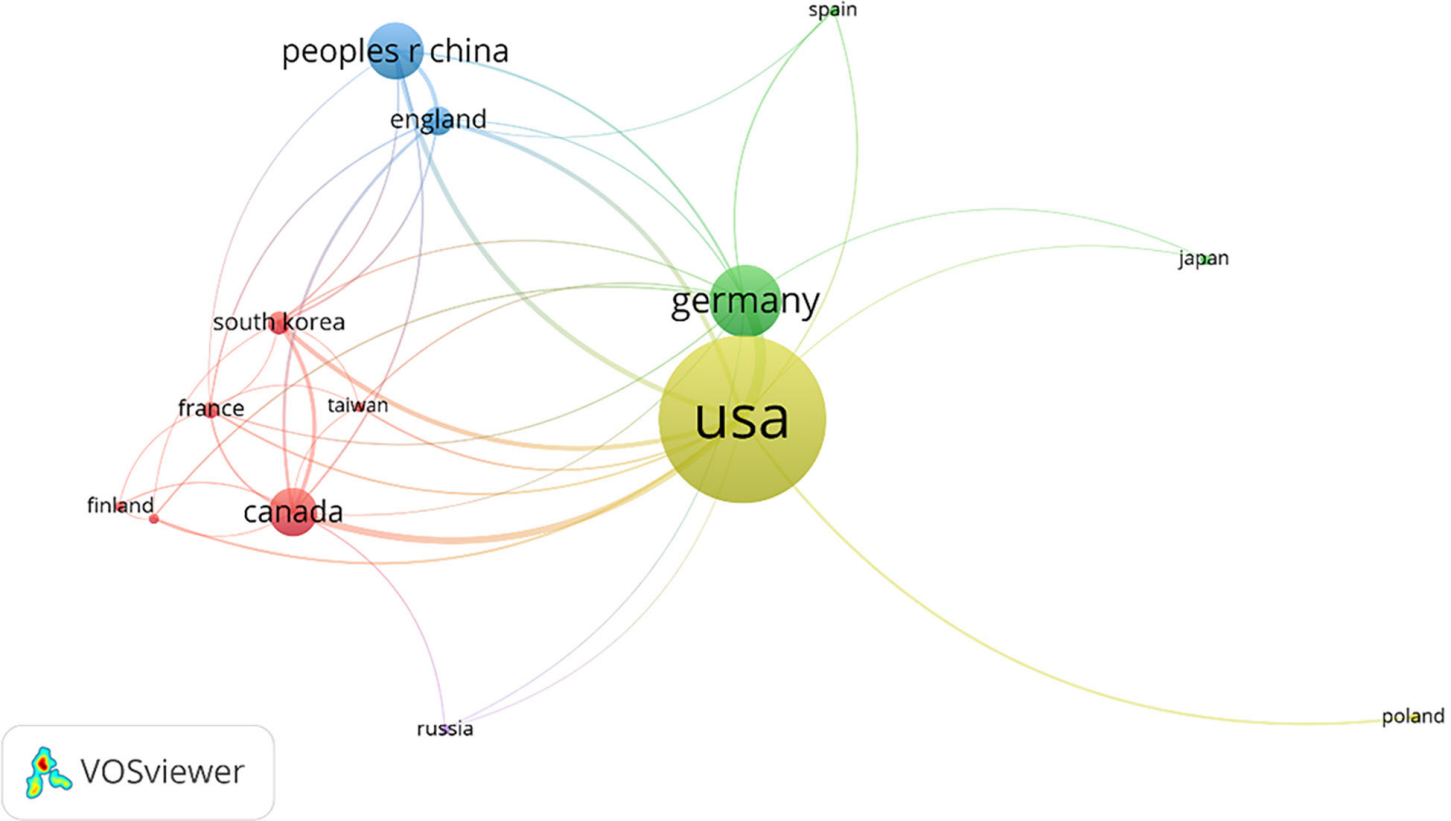
Co-authoring relationship Network map of countries’ co-authoring relationships. In total, 14 countries showed a co-authoring relationship.

A country’s minimum posting volume was limited to 5, and a total of 14 countries were screened out of the 31 countries as having cooperative relations. The United States has close cooperative relations with most countries (**Figure 9**).

## Discussion

As a promising gene therapy agent, vaccinia oncolytic virus is considered as an ideal cancer gene therapy drug in the future, and has been applied in clinical trials (13). In recent years, the number of studies in this research field has been increasing year by year, and the cooperation between authors and institutions in various countries has become closer, which makes relevant bibliometrics and quantitative analyses of the literature particularly important. In this study, we used Histcite Pro and VOSviewer to perform quantitative and visual analyses on 408 relevant research articles from 2002-01-01 to 2022-04-01, obtained from Web of Science through a keyword search.

The number of publications is the most significant indicator of whether and when a field is a research hotspot. The years 2013 and 2020 exhibited the two peaks in publication volume. Before 2013, the research in this field was still in the preliminary stage, but the annual publication volume had been rising steadily, indicating that vaccinia oncolytic virus has been gradually entering people’s consciousness since 2002. In 2013, the publication of the “Randomized dose-finding clinical trial of oncolytic immunotherapeutic vaccinia JX-594 in liver cancer”, ranked fourth in terms of LCS, attracted the attention of the academic community. The team used JX-594 on patients with advanced liver cancer, and they not only demonstrated that the dose was an important determinant of overall survival in subjects with advanced cancer, but this was also the first randomized clinical trial to show that an oncolytic virus or gene therapy drug can be associated with a significant improvement in overall survival (9). In the following few years, research began to develop rapidly, and although the number of publications from 2013 to 2019 decreased slightly, it was generally much higher than before 2013. This shows the strong interest in this emerging field of research. The United States is far ahead of the rest of the world in terms of the number of posts, followed by Germany, with the two countries combined contributing more than half of the total number of posts. The strains used in the current research on oncolytic vaccinia virus include Wyeth, Western Reserve, Copenhagen, Lister, and Tian Tan strain (2). Among them, the oncolytic vaccinia viruses approved for clinical research include GLV-1h68 (Lister strain) and jx-594 (Wyeth strain), which are widely used in many countries, such as the United States, Germany, etc. Szalay AA is the most published expert in the field of oncolytic viruses, and has published 75 related core papers. He has performed in-depth research on oncolytic viruses and immunology (14), and its clinical applications (15).

LCS refers to the number of times a document has been cited in similar articles. A higher LCS reflects a higher degree of recognition of the research results of the article in the peer field (16). GCS refers to the number of times the study is cited in all fields. The H-index is generally used to evaluate the quantity and level of the academic output of researchers, and can generally be used to measure the impact of papers (17). Therefore, the LCS, GCS and H-index are all important indicators used for evaluating academic quality. We found that the United States takes the leading position in terms of both the LCS and H-index, and the top ten articles in terms of LCS were all contributed by American institutions or authors, which shows that the research influence of the United States in this field is extremely high. The emergence of this phenomenon may be due to the international recognition of their in-depth research and clinical experiments on classic oncolytic viruses such as JX-594 (9, 18, 19). Interestingly, most of the top 10 studies in terms of LCS were published by multi-country and multi-institutional collaborative entities, which reflects the extensive international cooperation and consensus in the area of vaccinia oncolytic virus research, and the research fields tend to be diversified, thus strengthening various fields. Cooperation in this regard may yield new results.

We found that among the authors in the field, the findings of BELL JC and Szalay AA were the most subject to peer consensus, and ranked highest in terms of quantity, LCS, and H-index. This shows that the two scholars have a more in-depth and comprehensive understanding of this field. In particular, Szalay AA has undertaken significant research in this area, and relevant scholars can cooperate with his team if they want to partake of this knowledge. Of the top ten publications, Univ Wurzburg and Genelux Corporation have performed the most and deepest research on vaccinia oncolytic virus. Experts who want to study such topics can contact these two institutions. Looking at the top ten articles in terms of LCS, we found that four of them were published in “MOLECULAR THERAPY”; interested scholars can consider publishing new research results in this field in this journal. The journal’s 2021 impact factor was 11.454 points. It is an internationally leading journal for the study of using molecular and cell therapy to correct genetic and hereditary diseases, and shows that vaccinia oncolytic virus has received extensive attention in the field of molecular and cell therapy.

The main keywords in the study field of vaccinia oncolytic virus are cancer (red), therapy (treatment, blue), jx-594 (modified vaccinia virus, yellow), and immunotherapy (immunotherapy). The red area concerns the study of the mechanism of action of vaccinia oncolytic virus in tumor cells; the green part concerns the study of the changes of immune response and tumor microenvironment after vaccinia oncolytic virus infection; the blue part concerns the treatment of tumors with vaccinia oncolytic virus. As regards the research using animal experiments, the yellow part is the research direction of the clinical application of vaccinia oncolytic virus in the treatment of tumors. Early studies mainly focused on animal experiments. A representative study is “Eradication of solid human breast tumors in nude mice with an intravenously injected light-emitting oncolytic vaccinia virus”, published by Zhang Q et al. in 2007. The applicability of oncolytic vaccinia virus in eradicating breast tumors was proven by animal experiments (20). This suggests that the mechanism of oncolytic virus-induced tumor eradication may be a combination of immune activation and viral oncolysis, which provides some ideas for the design of oncolytic virus therapy in the future. The focus of mid-term research tends to be diversified, and research on the mechanism and the impact of vaccinia oncolytic virus on the tumor microenvironment is gradually emerging. In recent years, with the gradual development of molecular biology, virology, and genetics, in addition to the above problems, studies on suicide gene, jx-594 (genetically modified vaccinia virus), and combination drugs have gradually increased. The current research on jx-594 has been relatively in-depth, and more than 40 relevant papers have been published since the beginning of 21st century. Therefore, we speculate that the use of vaccinia oncolytic viruses to develop suicide genes may be a future research hotspot. Suicide genes, also known as drug-sensitive genes, were first studied in 2008. Foloppe J et al. constructed a thymidine kinase deletion vaccinia virus (Copenhagen strain) expressing the FCU1 gene for use in colon cancer (21). This research has not progressed significantly in the years since. TG6002 represents a breakthrough. Through the expression of FCU1 combined with the targeted deletion of the J2R and I4L genes, stronger tumor selective replication and tumor cell killing can be achieved than with the previous vaccinia virus carrying a suicide gene alone (22, 23). In addition, in recent years, Jeong SN et al. designed a novel vaccinia oncolytic virus carrying a tumor suicide gene and a normal angiogenesis gene. This novel vaccinia oncolytic virus can successfully induce apoptosis in tumor-targeted cells, and generate anti-tumor immunity (24). Therefore, we speculate that the design of a more versatile vaccinia oncolytic virus carrying a suicide gene may be a future development direction. In addition to suicide genes, the use of the chemokines expressed by the vaccinia oncolytic virus to kill tumors is also a common approach of gene therapy (25). The chemokines expressed by oncolytic viruses can specifically attract T cells to the tumor, thus inducing a strong systemic anti-tumor immune response and significantly enhancing the efficacy of oncolytic virus therapy. Scholl SM has been using recombinant vaccinia viruses encoding human MUC1 and IL2 as an immunotherapy for breast cancer patients for more than 20 years, beginning in 2000 (26). In recent years, some combined gene therapy approaches, such as the combination of jx-594 and ipilimumab for melanoma and IL-24 and GM-CSF for liver cancer, have also been gradually developed (27, 28).

At present, the development of new vaccinia oncolytic viruses, such as recombinant oncolytic viruses that block immunosuppressive checkpoints and activate immune co-stimulatory signaling pathways, and recombinant oncolytic viruses that combine multiple immune effects, has become a popular research direction nationally and internationally. We also conducted a VOSviewer visual analysis of the co-cited references, co-cited scholars, and co-authorship relationships, and found that the United States ranks first in terms of cooperation between countries, cooperation among institutions, and co-authorship relationships. The United States undoubtedly dominates the research and development in this field.

Although bibliometrics and knowledge graph analyses offer retrospective summaries of research, they can also predict project future, cutting-edge issues. However, our research still has certain limitations. First, our study is limited to English publications listed on the Web of Science database, which may cause a small degree of bias in our research. Secondly, our research spans nearly 20 years, and there is thus a certain lack of comprehensiveness. However, if we increase the time span of our research too much, the predictability of future fields may decrease. With the advancement of science and technology and the development of medicine, the accuracy of bibliometrics and knowledge graph analyses will continue to improve, and we may thus conduct similar retrospective studies on other types of oncolytic viruses in the future.

## Data availability statement

The original contributions presented in the study are included in the article/supplementary material. Further inquiries can be directed to the corresponding authors.

## Author contributions

LB and LT have contributed equally to this manuscript. SH and MB are corresponding authors. All authors contributed to the article and approved the submitted version.
